# Emotion Dysregulation in Children with ADHD: Irritability is Associated with EEG-Measured Frontal Alpha Asymmetry in Response to Emotional Stimuli

**DOI:** 10.1007/s10802-026-01439-w

**Published:** 2026-03-25

**Authors:** Nastassia J. Hajal, Giulia Salgari, Sandra K. Loo

**Affiliations:** Department of Psychiatry and Biobehavioral Sciences, Semel Institute for Neuroscience & Human Behavior University of California, Los Angeles, CA U.S.A.

**Keywords:** ADHD, Irritability, Emotion, Children, EEG

## Abstract

A growing body of theoretical and empirical work supports an updated conceptualization of ADHD that includes emotion dysregulation as either a core feature or a specific subtype of the disorder; much of this work emphasizes emotions that are high in approach orientation, such as anger and high positive affect. This study sought to contribute to this body of work by examining relations between child- and parent-reported irritability and EEG biomarkers of emotion-related processing in 8- to 12-year-old children diagnosed with ADHD. We examined frontal alpha asymmetry (FAA), a neurophysiological index of the action tendency component of emotions, in response to emotionally evocative images, from 50 children. As expected, greater relative left FAA (reflecting greater approach tendency) in response to emotionally evocative (fearful and happy) stimuli was associated with higher irritability symptom levels. Early FAA (0-200 ms post-stimuli) was more strongly related to irritability than later FAA (250–600 ms post-stimuli), which supports the concept of emotional impulsivity. Furthermore, FAA findings seemed to be driven by lower alpha power over the left hemisphere, suggesting greater approach (vs. withdrawal) among those with high irritability. Overall, findings were consistent with previous research showing the impact of emotions in ADHD, and specifically of dimensions of irritability/anger-proneness and high positive affect. Findings build upon previous research by showing support at the neurophysiological level, and highlighting the importance of approach-related emotions, regardless of valence.

## Introduction

ADHD is one of the most commonly diagnosed disorders of childhood (Danielson et al., 2024), with many negative effects being sustained into adulthood (Barbaresi et al., 2013; Biederman et al., 2010, 2012). It is well-established that many children with ADHD show high levels of emotional dysregulation, especially anger dysregulation (see review by Faraone et al., 2019). Over the past decade, some scholars have argued for an updated conceptualization of ADHD that includes emotion dysregulation as either a core feature (Barkley, 2015) or a specific subtype (Karalunas et al., 2019; Nigg, Karalunas, Feczko et al., 2020) of the disorder. Improving our understanding of the nature of emotional processing in children with ADHD is a key foundational step towards improving outcomes for children with ADHD, and particularly those who may not respond to standard treatments. The vast majority of previous work on emotion in ADHD has focused on anger (including other emotions within the anger family, such as irritability and rage; Faraone et al. 2019; Nigg et al. 2020a, b), and suggests that a tendency to experience heightened levels of anger is common in children with ADHD and associated with poorer outcomes in a variety of domains. For example, one study showed that 91% of children with ADHD reported at least one symptom of irritability (Eyre et al., 2017), and at least two separate samples have identified an irritable subtype based on temperament characteristics (Goh et al., 2020; Karalunas et al., 2014). These studies suggest that children with ADHD and increased irritability have poorer outcomes than their less-irritable counterparts, including more significant impairment in daily life and greater likelihood of future onset of other psychological disorders (Eyre et al., 2017; Karalunas et al., 2019). Longitudinal research indicates that the irritable subtype is stable across 3 years; in fact, the ADHD irritable profile has stronger long-term stability than other disorders characterized by irritability, such as DMDD (Karalunas et al., 2019). Importantly, identifying specific subgroups of children with ADHD based on emotional characteristics appears to add new information to our understanding of these children, and is *not* simply reflective of co-morbid mood or behavioral disorders. For example, Karalunas et al. (2019) found that the ADHD irritable subtype was not simply redundant with comorbid ADHD and ODD or DMDD. In another large sample of children with ADHD, irritability/anger was associated with a measure of ADHD genetic risk, but not mood disorder-oriented genetic risk (Nigg et al. 2020a, b).

Previous research has also identified dysregulation of positive affect in some children with ADHD. In addition to the irritable temperament subgroup of ADHD described above, several studies have found a “surgent” temperament subtype, which is characterized by high-intensity positive affect, high levels of affiliation, assertiveness and activity, and low levels of shyness (Goh et al. 2020; Karalunas et al. 2014, 2019; Nigg et al. 2020a, b). While typically developing children show greater parasympathetic activity during a negative emotion induction task than a positive emotion task, children with ADHD show similar elevations in parasympathetic activity during positive and negative emotions (Musser et al., 2011, 2013). Similarly, an event-related potential study found that children with ADHD showed enhanced P1 (neural index of early attentional processing) to positive emotional stimuli relative to neutral stimuli—this effect was not found for children without ADHD (Karalunas et al., 2020). Some studies have shown that children with ADHD and high positive affect have higher rates of co-morbid psychopathology (Karalunas et al., 2019; Okado & Mueller, 2016), although this finding is not as robust as for ADHD + irritability.

Although anger and positive affect are typically thought of as dissimilar emotional states due to their differing valence, they share the same *action tendency* (i.e., readiness to engage with emotion-eliciting stimuli, or, approach/withdrawal), which is a core component of some theories of emotion. For example, functional emotion theory conceptualizes specific emotional states (e.g., anger, fear, joy) partly in relation to an individual’s action tendency (Frijda, 1988; also see Barrett & Campos, 1987 for a developmental perspective and Keltner & Gross, 1999 for a review). From a functional perspective, anger and (high) positive affect are both approach-oriented emotional states. The relevance of approach/withdrawal for ADHD has previously been recognized in work on temperament (Nigg, 2006) and behavioral inhibition and activation systems (Luman et al., 2012; Quay, 1997) and motivational models (Barkley, 2015; Mitchell, 2009). Studies of emotion and ADHD that have probed the effects of broad positive and negative emotion factors support the idea that approach- (versus withdrawal-) oriented emotions play a unique role. For example, Karalunas et al. included non-approach emotions in their estimation of a negative affect subtype (fear and sadness), but found that it was anger in particular that contributed to the poorer clinical outcomes seen in this group (Karalunas et al., 2019). It is also notable that the surgent subtype identified in several studies of children with ADHD includes only high-intensity positive affect characterized by approach (e.g., excitement, joy); in these studies, low-intensity positive emotions characterized by withdrawal or neutrality (e.g., contentment) generally load onto a separate factor (Goh et al. 2020; Karalunas et al. 2014, 2019; Nigg et al. 2020a, b). Taken together, the large body of findings linking ADHD with irritability/anger and positive emotion suggests a unique significance of approach-oriented emotions in ADHD, *irrespective of valence*. This has important implications for assessment and characterization of emotion dysregulation in ADHD, in that researchers and clinicians must be careful to assess for the motivational component (i.e., action tendency, or, approach/withdrawal) of emotions, in addition to more commonly assessed “negative affect” and “positive affect.”

Another important theoretical construct in work on emotion and ADHD is that of emotional impulsivity, symptoms of which include “impatience, low frustration tolerance, hot-temperedness, quickness to anger, irritability, and easily emotionally excitable” (Barkley & Fischer, 2010, p. 503). Key to the conceptualization of emotional impulsivity is timing, and specifically, the quickness with which the emotion is experienced (Barkley, 2015). Because individuals with emotional impulsivity react so quickly, these emotions are less likely to be unconsciously downregulated, which then compromises opportunities for later conscious, effortful emotion regulation strategies. The concept of emotional impulsivity is also compatible with functional emotion theory, which connects the action tendency component of emotions to impulsive as well as deliberate behaviors; it essentially views impulsive behaviors as unmodulated action tendencies (Frijda, 2010). Thus, functional emotion theory may be a helpful unifying approach for integrating some of the key emotional constructs that have been identified in the ADHD literature.

### Psychophysiological Approaches to Studying Emotion in Children with ADHD

Some aspects of emotion occur so automatically and quickly that they are outside of conscious awareness; this may be especially true for individuals with ADHD given their vulnerability for emotional impulsivity. As a result, it is important to use multiple methods to measure emotion-oriented processes in individuals with ADHD so as not to rely exclusively on self-report of subjective emotional states. This is especially true for children, whose developmental capacity renders them less able to conceptualize and verbalize emotional states.

Electroencephalogram (EEG) methods are particularly useful in studying emotion because EEG (1) indexes neurophysiological responses on an extremely fast time-scale (faster than other imaging methods, like fMRI), enabling capture of immediate responses to stimuli that may not be available to awareness, and (2) a large literature shows that hemispheric asymmetry in EEG-measured alpha power over the frontal cortex is associated with the motivational component of emotion, or action tendency. In both adults and children, greater right- relative to left-hemisphere alpha band (8–13 Hz [Hz]) activity (or, right frontal alpha asymmetry; FAA) is associated with avoidance and withdrawal, while greater left FAA is associated with approach (Reznik & Allen, 2018). Although EEG generally has coarse spatial resolution, studies that have used advanced signal processing analyses (including one using a sample of youth with ADHD), have localized EEG-measured frontal alpha asymmetry to the dorsolateral prefrontal cortex (DLPFC; Auerbach et al., 2015; Ellis et al., 2017; Pizzagalli et al., 2005). The DLPFC is known to regulate key limbic structures involved in the reward system (Ballard et al., 2011), which involves approach motivation and behavior, as well as the amygdala (Ray & Zald, 2012), which is involved in the fight/flight/freeze response. Specifically, the DLPFC initiates signals to the mesolimbic structures that release dopamine (ventral tegmental area, nucleus accumbens) to initiate motivated behavior (Ballard et al., 2011), and right DLPFC has been shown to disrupt reward sensitivity in approach-avoidance situations (Rolle et al., 2022).

EEG-measured FAA is associated with both observed and self-reported behaviors, as well as with approach- versus avoidance-oriented emotions. For example, increases in left FAA in the context of frustration-inducing experimental paradigms (e.g., participants are insulted or rejected), are associated with self-reported anger as well as increased behavioral aggression (Harmon-Jones & Sigelman, 2001; Jensen-Campbell et al., 2007; Verona et al., 2009). Conversely, increases in right FAA in the context of sadness or fear inducing paradigms are associated with avoidance (Pérez-Edgar et al., 2013), self-report of sadness and anxiety (Killeen & Teti, 2012), and longer duration of sad and tense facial expressions (Hajal et al., 2017). In addition to studies examining *changes* in FAA in response to a stimulus, others examining FAA at rest show that it is related to approach and withdrawal in their more enduring forms; for example, resting left FAA is associated with temperamental behavioral activation, trait anger, and capacity for distress tolerance, while resting right FAA is associated with temperamental behavioral avoidance and withdrawal-oriented disorders like depression (Ellis et al., 2018; Harmon-Jones & Gable, 2018; Reznik & Allen, 2018). In sum, EEG-measured FAA can differentiate specific action tendencies associated with discrete emotions, including those most relevant to ADHD (i.e., anger, irritability, and surgency).

Existing studies examining EEG-measured FAA in ADHD have shown mixed findings, with some reporting that ADHD is associated with greater right asymmetry, others reporting greater left asymmetry, and still others reporting no relation (see review in Alperin et al., 2019). It may be that mixed results are due to variation in emotional symptoms associated with ADHD, which are not always accounted for in analyses. Alperin and colleagues (2019) showed that there were no differences in resting FAA based on ADHD diagnosis alone, but were when level of negative affect was taken into account. Further, for those studies that *do* examine emotional characteristics, the majority of studies test a general negative affect construct that confounds approach-oriented negative emotions (namely anger) with withdrawal-oriented negative emotions (like sadness and fear).

Another gap in the (albeit limited) work on FAA and ADHD is that most studies have examined resting FAA as opposed to changes in FAA in response to an emotionally evocative stimulus or task. Yet, non-ADHD focused studies demonstrate that asymmetry measured during an emotional challenge is more predictive of actual emotional responding than resting asymmetry; this is likely because state-related changes in asymmetry may reflect capacity for emotion regulation (Coan et al., 2006). Several studies have shown that task-induced FAA is more predictive of a variety of outcomes than resting FAA, including depression (Stewart et al., 2014) and facial expressions of emotion (Hajal et al., 2017). Furthermore, there is evidence that more individual variation exists in task-induced FAA than resting FAA, and that resting FAA is more vulnerable to measurement error (Coan et al., 2006). One of the few studies that has examined state-related FAA in youth with ADHD showed support for the notion that approach-related processes in individuals with ADHD are associated with FAA. Specifically, youth with ADHD showed greater left FAA (reflecting greater approach motivation) during trials of a go-no go task in which they failed to inhibit a response; this relation was not found for typically developing participants (Ellis et al., 2017).

Additionally, most studies of FAA have not taken advantage of the fine time resolution of EEG; even those that examine task-induced changes in FAA often collapse the EEG data across a number of seconds (Ellis et al., 2017). Yet, the time resolution of EEG can provide important information related to emotionality in ADHD, given the centrality of timing to the conceptualization of emotional impulsivity (Barkley, 2015). Ellis and colleagues (2017) showed that children with ADHD were differentiated from children without ADHD only during the early stages of processing (Ellis et al., 2017). EEG studies that have used measures other than FAA also provide support for the importance of sensitivity to timing; for example, an ERP study of reactions to happy, fearful, and neutral emotion expressions found differences between children with and without ADHD only for early components (P1), but not later components (late positive potential; Karalunas et al., 2020). The fact that EEG indexes emotional processes at such a fast time-scale, *and* that there is a robust literature linking FAA to emotion and action tendency, make it a valuable method to examine emotion in children with ADHD.

The current study aimed to characterize brain-based correlates of emotion processing, emotional symptomatology reported by parents and children, and psychiatric comorbidity in ADHD. Specifically, it examined whether irritability in ADHD is associated with a neurophysiological marker of approach. In a sample of 8- to 12-year-olds with ADHD, we assessed FAA in response to emotionally evocative and neutral stimuli, and examined associations of FAA with reports of irritability symptoms (approach-oriented, negatively-valenced).

We expected children with higher levels of irritability symptoms to demonstrate a general propensity toward approach-oriented activation in response to emotionally evocative stimuli, as well as emotional impulsivity. Specifically, we hypothesized that irritability symptoms would be associated with greater relative left FAA (1) during emotionally evocative (fearful and happy), but not calm, conditions, and (2) during early (0–200 ms) but not late (250–600 ms) periods post-stimulus, implicating emotional impulsivity. Additionally, we hypothesized that children with clinically significant levels of irritability symptoms would show greater left FAA during emotionally evocative (fearful and happy) stimuli than children without clinically significant levels of irritability symptoms.

### Method

#### Participants

The current analysis draws on baseline data from a randomized controlled trial of a non-medication treatment for pediatric ADHD. Families of 8- to 12-year-olds were recruited via online and community advertisements. Eligibility criteria included meeting diagnostic criteria for ADHD based on several study-administered measures: clinical interview, parent-report on the Kiddie Schedule for Affective Disorders and Schizophrenia (Kaufman et al., 1997), a score of > 24 on the clinician-administered ADHD Rating Scale (DuPaul et al., 1998), and a severity score ≥ 4 on the CGI (Guy, 1976). Children also had to demonstrate a full-scale IQ of ≥ 80 (based on Wechsler Abbreviated Scales of Intelligence subtests; Wechsler, 1999), be able to tolerate EEG and other study procedures, and not meet exclusion criteria (current major depression, autism spectrum disorder, lifetime psychosis, mania, seizure disorder, head injury with loss of consciousness, or baseline suicidality). A total of 62 children were enrolled into the study; the current manuscript uses data from 50 children who had usable baseline data for EEG and relevant questionnaire measures. Table 1 displays demographic and mental health characteristics of the current sample.

**Table 1:** Demographic and mental health characteristics of study sample

	*N*	Mean	Std. Deviation	Minimum	Maximum
**Gender**					
Male	33				
Female	17				
**Ethnicity**					
Hispanic or Latino	9				
Not Hispanic or Latino	41				
**Race**					
White	34				
Black or African American	4				
Asian	7				
Other	5				
**Age (years)**	50	10.41	1.39	8.07	13.00
**Anger Dysregulation/Irritability Symptoms**					
Child-report ARI	48	4.27	2.86	0	10
*n* meeting clinical cutoff	33				
Parent-report ARI	50	4.26	3.83	0	12
*n* meeting clinical cutoff	23				
**Depressive Symptoms**					
Child-report CDI	50	9.14	5.68	0	22
*n* meeting clinical cutoff	3				
**Severe Mood Dysregulation and/or ODD**					
*n* meeting clinical cutoff	14				

### Procedure

A full description of methods and procedures for the study is available in McGough and colleagues (McGough et al., 2019). The study was approved by the UCLA Institutional Review Board. Regarding informed consent, both parents and children received thorough verbal and written descriptions of study requirements. Parents provided written permission for their child to participate, and children provided assent. The current analysis uses EEG and questionnaire data from the baseline assessment of the study only.

The baseline assessment was conducted at the laboratory, and included administration of the K-SADS, parent and child completion of the Affective Reactivity Index (ARI), and the EEG assessment. The EEG assessment included a resting baseline task, a spatial working memory task, a flanker task, and the Happy Café task. The current analysis used data from the Happy Café Task, which was adapted from previous use in fMRI studies (e.g., Miklowitz et al., 2017). During the task, participants viewed images from a standardized stimulus set (NimStim) of adults expressing fearful, calm, and happy facial expressions. Participants were instructed to press the left mouse key when they saw a picture of a girl, and the right mouse key when they saw a picture of a boy. Each trial lasted until the participant gave a response, or until 2000 ms had passed; whichever came first. Trials were separated by a 250 ms interstimulus interval during which a fixation cross showed on the screen. The task contained one block of 96 trials that repeated, for a total of 192 trials. Images were presented and responses captured in E-Prime.

EEG data were collected with an Electrical Geodesics 128-Channel Hydrocel Cap (EGI, Eugene, OR) and recorded in Netstation. During recording, data were referenced to Cz with a sampling rate of 1,000 and an impedance threshold of 50k ohms. Ocular movements were captured with electrodes at the outer canthus of each eye (for horizontal movements) and above the eyes (for vertical movements). After recording, data were exported into EEGLab for pre-processing and cleaning. First, data were high-pass filtered (> 1 Hz), re-referenced to average (excluding noisy channels and epochs). Then, data were decomposed with independent components analysis (ICA), which statistically separates brain from non-brain (e.g., ocular artifacts, muscle movement, environmental noise) data. Components identified as representing non-brain activity were rejected, and remaining data were back-projected into channel space for analysis. EEGLab was used to create event-related spectral perturbation (ERSP) plots that captured millisecond-level change in spectral power following the onset of a stimulus. Alpha power (8–12 Hz) was extracted from two frontal electrodes: F3 on the left side of the head and F4 on the right for each child.

### Measures

#### Symptoms

Irritability was measured with the Affective Reactivity Index (ARI), completed individually by child and parent participants. The ARI is a questionnaire that obtains ratings for six questions about irritability on a 0 (not true) to 2 (certainly true) Likert scale. The scale, which is widely used in both clinical and research settings, has well-established reliability and validity in youth samples, including good internal consistency for both youth- and parent-report forms and associations with measures of internalizing and externalizing symptoms and emotion regulation (Evans et al., 2020). Previous research has also established clinical cutoffs for the ARI (child-report scores > 2, parent-report scores > 3; Mulraney et al., 2014).

Diagnostic criteria for oppositional defiant disorder (ODD) and severe mood dysregulation (SMD) were assessed via the K-SADS (Kaufman et al., 1997).

#### Frontal Alpha Asymmetry (FAA)

Extracted alpha (8–12 Hz) power data were averaged across two time periods after each stimulus presentation: 0–200 ms (early) and 250–600 ms (late), for each stimulus type (Fear, Happy, Calm). In order to create an asymmetry score, alpha power measured from the left frontal electrode (F3) was subtracted from the right frontal electrode (F4; see Reznik & Allen, 2018). In keeping with the larger FAA literature, we considered greater FAA scores to reflect greater approach tendency (Reznik & Allen, 2018).

### Analytic Approach

Analyses were conducted in SPSS version 30 and R version 4.4.1. First, we examined descriptive statistics, and used repeated measures ANOVA to examine whether there were differences in FAA by emotion condition (calm, happy, fear), both for the full sample and when the sample was split into two groups based on the ARI clinical significance threshold. In addition to examining statistical significance, we evaluated effect size using partial eta squared (η²p), using commonly cited thresholds to determine magnitude (small = 0.01, medium = 0.06, large = 0.14; Cohen, 2013; Richardson, 2011).

Given that this small sample provides low power to detect effects, particularly when it is broken down into subgroups, we further examined associations between FAA and ARI as a continuous variable. Specifically, multiple regression analyses were used to test hypotheses that irritability symptoms would be associated with greater relative left FAA (1) during emotionally evocative (fearful and happy), but not calm, conditions, and (2) during early (0–200 ms) but not late (250–600 ms) periods post-stimulus. To guard against Type 1 errors, we adjusted *p*-values for a priori hypothesized relations using the Benjamini-Hochberg false discovery rate for the models in which we expected to see statistically significant associations (models assessing FAA during the early period for happy and fearful conditions, for both child- and parent-report; 4 models total). For models in which we expected to see differences in effects across emotion conditions (i.e., calm versus emotionally evocative conditions) or time periods (i.e., early versus late post-stimulus periods), we tested the differences in beta weights by calculating z-scores of the difference.

Follow up, exploratory regression analyses were then conducted to determine the impact of: (1) entering alpha power at F3 and F4 into the models in place of the asymmetry difference score, and (2) adding ODD/SMD diagnoses to the models. Finally, we used ANCOVAs to examine whether mean levels of FAA during emotionally evocative (fearful and happy) stimuli differed between children with ADHD *plus* clinically significant levels irritability and those with ADHD only. We did not employ any *p-*value adjustments for these follow-up analyses, given their exploratory nature.

All analytic models controlled for child gender and age.

## Results

### Descriptive Statistics

We started by examining descriptive statistics among variables of interest. For EEG variables, we examined descriptive statistics for alpha power at F3 and F4 separately, as well as the traditional EEG asymmetry (i.e., FAA) difference score (see Table 2). Child- and parent-reports of the ARI were significantly correlated; *r* =.54, *p* <.001.

**Table 2:** Descriptive statistics for EEG variables

	*N*	Minimum	Maximum	Mean	Std. Deviation	Skewness
**Fear Condition**						
Frontal alpha asymmetry (FAA 0–200)	50	−1.98	2.53	−0.08	0.90	0.31
F4 alpha 0–200 ms	50	−3.64	2.28	−0.09	1.03	−0.69
F3 alpha 0–200 ms	50	−3.04	2.45	−0.01	1.13	−0.51
FAA 250–600	50	−1.30	3.20	0.18	0.88	0.79
F4 alpha 250–600 ms	50	−4.29	1.84	−0.53	1.36	−0.86
F3 alpha 250–600 ms	50	−3.73	1.24	−0.72	1.27	−0.52
**Happy Condition**						
FAA 0–200	49	−1.85	1.80	−0.10	0.77	0.25
F4 alpha 0–200 ms	49	−2.50	2.36	0.12	1.03	0.06
F3 alpha 0–200 ms	49	−2.79	3.25	0.21	1.09	−0.02
FAA 250–600	49	−2.24	2.18	−0.06	0.78	−0.07
F4 alpha 250–600 ms	49	−4.35	1.85	−0.54	1.37	−0.48
F3 alpha 250–600 ms	49	−4.52	1.89	−0.49	1.26	−0.74
**Calm Condition**						
FAA 0–200	50	−2.14	3.20	0.01	0.90	0.88
F4 alpha 0–200 ms	50	−2.09	2.26	0.18	0.95	0.14
F3 alpha 0–200 ms	50	−2.24	2.57	0.17	1.04	0.13
FAA 250–600	50	−3.50	3.02	0.31	0.98	−0.64
F4 alpha 250–600 ms	50	−4.01	1.52	−0.59	1.14	−0.68
F3 alpha 250–600 ms	50	−4.09	2.88	−0.90	1.39	−0.14

### FAA by Condition

#### Early (0–200 ms)

A repeated measures ANOVA indicated a statistically significant, medium quadratic effect across the three emotion conditions (calm, happy, fear) from 0 to 200 ms after stimulus onset, *F*(1, 46) = 4.88, *p* =.032, η²*p* =.10 (model controlled for child age and gender). Visual inspection of the means indicated that FAA was more right lateralized for the happy (*M* = − 0.10, *SD* = 0.77) and fearful conditions (*M* = − 0.10, *SD* = 0.90), and closer to zero (although still right lateralized) for the calm condition (*M* = − 0.05, *SD* = 0.78).

Two follow up repeated measures ANOVAs included a between-subjects factor indicating whether the child met the clinical significance threshold on the child-report ARI (*n* = 32) and parent-report ARI (*n* = 22). There was a medium, significant between-subjects effect of the child-report ARI, *F*(1, 43) = 5.66, *p* =.022, η²*p* =.12. The between-subjects effect for parent-report ARI was small and not statistically significant (*F*(1, 43) = 2.40, *p* =.128, η²*p* =.05), however, visual inspection of the means for both models indicated that children with clinically significant irritability showed *left* lateralized FAA for happy and fear conditions, in contrast to the right lateralization seen in the full sample and in children who did not have clinically significant ARI scores. FAA was similar for both groups during the calm condition.

#### Late (250–600 ms)

A repeated measures ANOVA indicated a medium but only marginally significant linear trend across the three emotion conditions (calm, happy, fear) from 250 to 600 ms after stimulus onset, *F*(1, 46) = 3.38, *p* =.072, η²*p* =.10 (model controlled for child age and gender). Visual inspection of the means indicated that, by 250–600 ms, FAA was more left lateralized for the fear (*M* = 0.21, *SD* = 0.86) and calm (*M* = 0.25, *SD* = 0.91) conditions, but close to zero for the happy condition (*M* = − 0.06, *SD* = 0.78). The follow up repeated measures ANOVAs that included a between-subjects factor for clinically significant ARI did not indicate significant between-subjects effects for parent or child report of the ARI (*p*s > 0.30).

### Association Between FAA and Irritability as a Continuous Variable

Tests of associations between FAA and irritability as a continuous variable can be found in Tables 3 and 4. Consistent with hypotheses, during the *Fearful* condition, early FAA (i.e., 0–200 ms post-stimulus) was associated with ARI scores for both child-report (β = 0.40, *p* =.006, 95% CI [0.12, 0.68]) and parent-report (β = 0.29, *p* =.038, 95% CI [0.02, 0.56]), such that greater relative left FAA (reflecting greater approach tendency) was associated with higher irritability symptom levels, regardless of reporter. These relations survived *p-*value correction for child-report (FDR adjusted *p* =.024) and became marginally significant for parent-report (FDR adjusted *p* =.051). Neither association was sustained into the later period of the *Fearful* stimulus presentation (i.e., 250–600 ms post-stimulus; all *p*s > 0.10), although beta weights were not significantly different between the early and late models for either child (β diff = 0.18, *z* = 0.86, *p =*.39, 95% CI [−0.23, 0.58]) or parent (β diff = 0.13, *z* = 0.65, *p =*.514, 95% CI [−0.26, 0.52**]**) report ARI.

For the *Happy* condition, results were consistent with hypotheses for child-report but not parent-report. Child-report of the ARI was significantly associated with early FAA (β = 0.38, *p* =.012, 95% CI [0.09, 0.67]), such that greater relative left frontal activity (reflecting greater approach tendency) was associated with higher irritability symptom levels; this relation survived *p-*value correction (FDR adjusted *p* =.024). For the later post-stimulus period (250–600 ms post-stimulus), this relation was in the same direction but reduced in magnitude, and only marginally significant (β = 0.27, *p* =.07, 95% CI [−0.03, 0.57]). Differences in beta weights between the early and late models were not significantly different, however (β diff = 0.11, *z* = 0.51, *p =*.61, 95% CI [−0.30, 0.51]). Parents’ report of the ARI was not related to FAA during either period of the *Happy* condition (all *p*s > 0.10).

As expected, FAA during the *Calm* condition was not associated with either child- or parent-report of the ARI (all *p*s > 0.10), however, tests of differences in β between calm conditions and emotionally evocative conditions were not statistically significant (child report early calm versus early fear β diff = − 0.34; *z* = −1.59, *p* =.11, 95% CI [−0.75, 0.08]; child report early calm versus early happy β diff = − 0.31; *z* = −1.47, *p* =.14, 95% CI [−0.73, 0.11]; parent report early calm versus fear β diff = − 0.12; *z* = − 0.57, *p* =.57, 95% CI [−0.51, 0.28].

**Table 3:** Regression Models Predicting Child-reported Irritability from Frontal Alpha Asymmetry

0–200 ms						250–600 ms					
Predictor	B	β	95% CI for β	SE	*p*	Predictor	B	β	95% CI for β	SE	*p*
**Calm**						**Calm**					
Age	0.047	0.023	[−0.284, 0.33]	0.313	0.882	Age	0.009	0.005	[−0.299, 0.308]	0.309	0.976
Gender	−0.03	−0.005	[−0.324, 0.314]	0.965	0.976	Gender	−0.077	−0.013	[−0.321, 0.295]	0.932	0.934
FAA	0.197	0.062	[−0.256, 0.381]	0.501	0.696	FAA	0.394	0.137	[−0.171, 0.445]	0.439	0.374
**Happy**						**Happy**					
Age	−0.106	−0.051	[−0.342, 0.239]	0.298	0.723	Age	−0.014	−0.007	[−0.304, 0.291]	0.306	0.964
Gender	−0.041	−0.007	[−0.293, 0.28]	0.887	0.963	Gender	0.087	0.014	[−0.283, 0.311]	0.919	0.925
FAA	1.446*	0.376*	[0.086, 0.666]	0.553	0.012^a^	FAA	0.998†	0.271†	[−0.026, 0.568]	0.542	0.072
**Fearful**						**Fearful**					
Age	0.093	0.045	[−0.236, 0.326]	0.287	0.748	Age	0.141	0.068	[−0.239, 0.376]	0.314	0.656
Gender	0.077	0.013	[−0.268, 0.293]	0.848	0.928	Gender	0.218	0.036	[−0.264, 0.336]	0.908	0.812
FAA	1.252**	0.397**	[0.117, 0.677]	0.437	0.006^b^	FAA	0.729	0.22	[−0.089, 0.528]	0.508	0.158

IV = independent variable

*p <.05; **p <.01; †*p* <.10

^a^ FDR-corrected *p* =.024

^b^ FDR-corrected *p* =.024

**Table 4:** Regression Models Predicting Parent-reported Irritability from Frontal Alpha Asymmetry

0–200 ms						250–600 ms					
Predictor	B	β	95% CI for β	SE	*p*	Predictor	B	β	95% CI for β	SE	*p*
**Calm**						**Calm**					
Age	0.823	0.299	[0.014, 0.584]	0.39	0.04	Age	0.752	0.273	[−0.015, 0.56]	0.394	0.062
Gender	−0.695	−0.087	[−0.387, 0.213]	1.194	0.563	Gender	−0.361	−0.045	[−0.338, 0.248]	1.167	0.759
FAA	0.748	0.175	[−0.123, 0.474]	0.633	0.243	FAA	0.301	0.077	[−0.215, 0.37]	0.567	0.598
**Happy**						**Happy**					
Age	0.655	0.238	[−0.05, 0.526]	0.393	0.103	Age	0.716	0.26	[−0.026, 0.547]	0.392	0.074
Gender	−0.552	−0.068	[−0.354, 0.218]	1.149	0.633	Gender	−0.402	−0.05	[−0.336, 0.237]	1.151	0.728
FAA	0.996	0.199	[−0.089, 0.487]	0.715	0.171^c^	FAA	0.821	0.168	[−0.117, 0.453]	0.693	0.242
**Fearful**						**Fearful**					
Age	0.837	0.304	[0.029, 0.579]	0.377	0.031	Age	0.89	0.323	[0.028, 0.618]	0.404	0.033
Gender	−0.297	−0.037	[−0.311, 0.237]	1.091	0.786	Gender	−0.134	−0.017	[−0.301, 0.268]	1.132	0.906
FAA	1.242*	0.29*	[0.017, 0.563]	0.58	0.038^d^	FAA	0.696	0.16	[−0.134, 0.455]	0.635	0.279

IV = independent variable

*p <.05; **p <.01; †*p* <.10

^c^ FDR-corrected *p* =.171

^d^ FDR-corrected *p* =.051

### Exploratory Analyses

#### Localization Analyses

To further probe the significant associations between irritability and frontal asymmetry, we re-ran the three regression models with FDR-adjusted *p-*values that were 0.05 or below, removing the FAA difference score and instead entering alpha power at F3 and at F4 as separate variables (controlling for child age and gender). This allowed us to examine differential contributions of data collected above each hemisphere. Results suggested that left (F3) alpha power was driving the association between FAA and irritability, regardless of condition or reporter. For the *Fearful* condition, early left (F3) alpha power—but *not* right (F4) power—was strongly associated with ARI scores, for both child (F3: βs = − 0.62, *p* =.001 95% CI [−2.46, −0.65]; F4: β = 0.25, *p* =.174, 95% CI [−0.32, 1.71]) and parent report (F3: β = − 0.45, *p* =.015, 95% CI [−2.76, −0.31]; F4: β = 0.20, *p* =.271, 95% CI [−0.60, 2.10]). The same general pattern held for child-report ARI and the *Happy* condition; only left alpha power was significantly associated with irritability symptoms (β = − 0.65, *p* <.004, 95% CI [−2.89, −0.59]), although there was also a trend-level association with right alpha power (β = 0.37, *p* <.083, 95% CI [−0.15, 2.25]).

#### Comorbid Irritability-related Diagnoses

In order to determine whether findings during Fearful and Happy conditions could be better accounted for by comorbid diagnoses of ODD or severe mood dysregulation (SMD), we again re-ran the models with FDR-adjusted statistically significant associations, this time including SMD and/or ODD diagnosis (binary variable) as a third covariate. SMD/ODD diagnosis was strongly related to child-report ARI at the bivariate level (*r* =.57, *p* <.001), but for all models run, several associations between irritability and frontal alpha remained significant. For the *Fear* condition, the associations between child- and parent-reported ARI and FAA reduced in magnitude; the association remained significant for child-report (β = 0.25, *p* =.046, 95% CI [0.02, 1.57]) but became nonsignificant for parent-report ARI (β = 0.16, *p* =.221, 95% CI [−0.42, 1.78]). For the *Happy* condition, the associations between child-report ARI and early FAA reduced in magnitude but remained significant (β = 0.26, *p* =.037, 95% CI [0.06, 1.95]).

#### FAA and Clinically Significant Irritability

We used ANCOVAs to examine whether mean levels of FAA differed between children with ADHD *plus* clinically significant levels irritability and those with ADHD only (controlling for child gender and age). We created high and low level symptom groups based on previously established clinical cutoffs for the ARI (child-report scores > 2, *n* = 32; parent-report scores > 3, *n* = 22; Mulraney et al., 2014).

For the *Fearful* condition, children with clinically significant ARI scores had higher FAA scores (reflecting greater approach motivation) than children without clinically significant ARI scores; this association was found for both child-report (*F*(1,44) = 4.85, *p* <.0335, η²*p* =.10) and parent-report (*F*(1,46) = 4.32, *p* =.043, η²*p* =.09) of the ARI.

For the *Happy* condition, children with clinically significant ARI scores had higher FAA scores (reflecting greater approach motivation) than children without clinically significant ARI scores, but only when examining child-report ARI (*F*(1,43) = 6.96, *p* =.012, η²*p* =.14); parent-report ARI results were nonsignificant (*F*(1,45) = 1.29, *p* =.262, η²*p* =.03).

## Discussion

ADHD is one of the most commonly diagnosed childhood mental health disorders, with negative impacts sustaining into adulthood (Barbaresi et al., 2013; Biederman et al., 2010, 2012). Despite the existence of several empirically supported medication and non-medication treatments for the core symptoms of ADHD (Cortese et al., 2018; Cortese & Coghill, 2018), standard treatments are less efficacious at improving other symptoms associated with ADHD (e.g., oppositionality, emotion dysregulation; Cortese et al., 2021) and significant numbers of children remain untreated (Danielson et al., 2024). In an effort to better understand etiological and maintaining factors of the disorder, in the past decade, there has been growing interest in a conceptualization of ADHD that includes emotion dysregulation as either a core feature (Barkley, 2015) or a specific subtype (Karalunas et al., 2019; Nigg, Karalunas, Feczko et al., 2020). Previous work on emotion in ADHD suggests a unique significance of emotions in the anger family (including irritability) and high intensity positive emotions. In this study, we conceptualize approach orientation as the unifying factor between findings on anger/irritability and high positive emotion, and tested the hypothesis that parent- and child-reported irritability in children with ADHD would be associated with a neurophysiological index of approach in response to emotionally evocative stimuli.

### Approach Orientation in Children with ADHD and Irritability

In line with hypotheses, greater child- and parent-reported irritability symptoms were associated with greater frontal alpha asymmetry (FAA; a neural index of approach-oriented activation) immediately after the onset (0–200 ms) of happy and fearful stimuli. This association was *not* found when viewing calm stimuli, which, in regard to approach orientation, would not be associated with either approach or withdrawal. Furthermore, during emotionally evocative conditions, the FAA difference score was driven primarily by alpha power measured over the left frontal cortex (as opposed to the right frontal cortex) which is consistent with previous research suggesting the unique impact of the left hemisphere on approach and related constructs (e.g., Pizzagalli et al., 2005).

FAA in response to happy stimuli, an approach-oriented emotion, was strongly associated with child-report of irritability. This suggests that the ARI, a measure of irritability, is to a large extent capturing general approach-orientation, regardless of emotional valence. This finding is in line with previous research that has found evidence for a “surgent” subtype of ADHD, which is characterized partly by temperamental high-intensity positive affect (Goh et al. 2020; Karalunas et al. 2014, 2019; Nigg et al. 2020a, b).

We predicted that children with high levels of irritability would show approach-oriented neural responses to emotionally evocative stimuli generally, including fear. Fear itself is a withdrawal-oriented emotion, and previous research shows greater relative right FAA in response to fear-based stimuli (Diaz & Bell, 2012; Pérez-Edgar et al., 2013) and in individuals who have psychological disorders characterized by fear, such as PTSD (Rabe et al., 2008). However, exposure to a fear expression in another person does not necessarily lead to fear in the person viewing the image. Furthermore, it is well-established that flight is not the only behavioral response associated with fear. “Freezing” and “fighting,” the latter of which is approach-oriented, are also possible responses to fear-based stimuli. The current findings suggest that children with irritability in addition to ADHD are more likely to have approach-oriented responses to emotionally evocative stimuli in general, including fear-based stimuli. This interpretation aligns with the evidence that children with higher levels of anger and aggression are more likely to hold an attribution bias that leads them to misinterpret ambiguous stimuli as having hostile intent (Verhoef et al., 2019). Future research should include angry stimuli as well as fearful stimuli to examine whether effects are stronger when emotion stimuli is clearly approach-oriented.

The current findings build upon previous research indicating a unique significance of approach-related emotions in ADHD, *regardless of valence*. This underscores the importance of emotion *granularity* (Barrett, 2017) in ADHD populations; in other words, considering *discrete* emotions such as happiness, calmness, contentment, sadness, anger, and fear, as opposed to the more commonly assessed “negative affect” and “positive affect.” The current findings suggest that discrete emotions may be associated with different brain states, and emotions may look mechanistically different depending on action tendency.

### Timing and Emotional Impulsivity

Another aspect of study findings that are promising and suggest the need for future research is related to the timing of FAA findings. Statistically significant associations were found for 0–200 ms after stimulus onset, *not* 250–600 ms. Although it is important to keep in mind that effects for early and late periods were not significantly different from *one another*, the general pattern of findings is consistent with the “emotional impulsivity” framework (Barkley, 2015) of approach-oriented emotion (particularly anger) in ADHD, which highlights the quickness and automaticity of the approach response. Similarly, an event-related potential study showed that, in contrast to typically developing children, children with ADHD had enhanced effects on *early* neural indices of attentional processing (P1 and N170), but not later processing (late positive potential) in response to positive emotional stimuli (Karalunas et al., 2020). Much of the extant research on emotion in ADHD focuses on trait-level emotionality; although important, measures of trait emotion are unable to capture temporal processes in emotion and ADHD (Karalunas et al., 2024), such as emotional impulsivity. Studies like the current one, and Karalunas et al., 2020, can play a key role in enhancing understanding of temporal dynamics of emotion in ADHD by using time-sensitive approaches to examining EEG data, which captures information on the order of milliseconds.

### Clinical Applications

The current findings may inform clinical practice with children with ADHD and irritability. They suggest ways that treatment might be customized for this population; e.g., including adjunctive emotion regulation training that helps children increase their awareness of angry or excited feelings and strategies to modulate them. They also help to inform understanding of the automaticity of approach-oriented emotions in children who have both ADHD and high levels of irritability, with differences in neural processing occurring even before cognitive processing of the stimulus. Providing education on the automaticity of irritable responding may prevent parents’ and caregivers’ attributions of children’s intentionality regarding impulsive angry behaviors, enabling parents to facilitate a more reparative parent-child interaction as opposed to escalating into a coercive cycle.

Study findings also suggest that EEG-measured alpha asymmetry may be a promising objective measure that could someday be incorporated into clinical assessments to inform treatment recommendations. For example, for a child with ADHD who shows high FAA in response to emotional stimuli, a clinician might recommend a cognitive behavioral or other emotion regulation based psychosocial intervention in addition to a psychostimulant medication. This may be particularly useful in cases where child self-report is unable to be obtained or is deemed unreliable. Although the lack of psychometric data and population-based norms for event-based FAA currently precludes its use in clinical assessment for psychological disorders, there has been some research on the reliability of the FAA signal during resting state (i.e., no stimulus condition; (Metzen et al., 2022) and some groups are working towards the development of norms for other EEG-measured signals (e.g., various ERP components; Imburgio et al., 2020), making its use in clinical practice a potential for the future.

## Limitations, Future Directions, and Conclusion

Several limitations of the current study should be addressed. First, the sample was small, and underpowered, particularly in the context of the number of statistical tests run. Although we did take steps to address these issues (conducting *p* value adjustments to reduce vulnerability to Type 1 errors, and conducting tests on beta weights to determine whether differences between significant and nonsignificant associations were meaningful), findings must be replicated with a larger sample before strong inferences can be made. The current sample was also relatively homogeneous in terms of race and ethnicity; future research in this area should aim to recruit samples that are diverse in terms of ethnoracial identity as well as socioeconomic status and other important demographic factors. Additionally, while the study design allowed us to disentangle approach- versus withdrawal-oriented emotions to a certain extent, the lack of anger stimuli was a limitation. Finally, while functional emotion theory emphasizes motivational orientation (approach/withdrawal), other theories of emotion consider arousal as a critical aspect differentiating discrete emotions (e.g., Bradley & Lang, 1994). Future studies on emotion in ADHD should include examination of parietal asymmetry, which research suggests is associated with the arousal component of emotion (Heller et al., 1997; Nitschke et al., 2000). Furthermore, even from the perspective of functional emotion theory, frontal alpha asymmetry cannot capture specific emotions, which are characterized by the specific combination of motivational orientation and appraisals (i.e., how attainable an individual perceives their goal for wellbeing).

Still, the current study adds to the literature in several ways. It contributes to the body of work supporting the inclusion of irritability as either a core feature or a subtype of ADHD. Importantly, it emphasizes the importance of considering motivational direction in this work, and provides neurophysiological support for this conceptualization. By making full use of the temporal resolution of EEG, it also provides additional support for the notion of emotional impulsivity in ADHD. Although specific clinical recommendations are premature, these findings set the stage for future research that can inform clinical assessment and treatment to improve outcomes for children with ADHD.

## Data Availability

Anonymized data may be available upon request to the author.
